# Haematological Alterations Associated with Selected Vector-Borne Infections and Exposure in Dogs from Pereira, Risaralda, Colombia

**DOI:** 10.3390/ani12243460

**Published:** 2022-12-08

**Authors:** D. Katterine Bonilla-Aldana, Erwin J. Gutiérrez-Grajales, Daniela Osorio-Navia, Mariana Chacón-Peña, Adrián E. Trejos-Mendoza, Soffia Pérez-Vargas, Lorenzo Valencia-Mejía, Luisa F. Marín-Arboleda, J. Paola Martínez-Hidalgo, María Angelica Reina-Mora, Luz Victoria González-Colonia, Jaime A. Cardona-Ospina, Erika Vanessa Jiménez-Posada, Diego Andrés Diaz-Guio, Jean Carlos Salazar, Manuel Sierra, Fausto Muñoz-Lara, Lysien I. Zambrano, Eduardo Ramírez-Vallejo, Juan Camilo Álvarez, Ingrid Lorena Jaramillo-Delgado, Samuel Pecho-Silva, Alberto Paniz-Mondolfi, Álvaro A. Faccini-Martínez, Alfonso J. Rodríguez-Morales

**Affiliations:** 1 Research Unit, Universidad Continental, Huancayo 12000, Peru; 2 Red Colombiana de Enfermedades Transmitidas por Garrapatas en Pequeños Animales (RECEPA)–Colombian Network of Tick-Borne Diseases in Small Animals (RECEPA), Pereira 660003, Colombia; 3 Institución Universitaria Visión de las Américas, Pereira 660003, Colombia; 4 San Lucas Centro Veterinario y Diagnóstico, Pereira 660003, Colombia; 5 Grupo de Investigación Biomedicina, Faculty of Medicine, Fundación Universitaria Autónoma de las Américas, Pereira 660003, Colombia; 6 Grupo de Investigación en Infecciones Emergentes y Medicina Tropical, Instituto para la Investigación en Ciencias Biomédicas, SCI-HELP, Pereira 660003, Colombia; 7 Division of Infectious Diseases and Vaccinology, School of Public Health, University of California, Berkeley, CA 94704, USA; 8 Vitalcare, Armenia, Quindío 630001, Colombia; 9 Unit of Scientific Research, School of Medical, Faculty of Medical Sciences, Universidad Nacional Autónoma de Honduras (UNAH), Tegucigalpa 11101, Honduras; 10 Department of Internal Medicine, Faculty of Medical Sciences, Universidad Nacional Autónoma de Honduras (UNAH), Tegucigalpa 11101, Honduras; Department of Internal Medicine, Hospital Escuela, Tegucigalpa 11101, Honduras; 11 IPS Cardiológica Eduardo Ramírez, Pereira 660003, Colombia; 12 Grupo de Investigación One-Health, Departamento de Investigación de Enfermedades Infecciosas en Animales, Centro de Diagnóstico Especializado Testmol, Medellín, Antioquia 050001, Colombia; 13 Faculty of Health Sciences, Universidad Cientifica del Sur, Lima 15046, Peru; 14 Hospital Nacional Edgardo Rebagliati Martins, Lima 15072, Peru; 15 Laboratory of Medical Microbiology, Department of Pathology, Molecular and Cell-based Medicine, The Mount Sinai Hospital-Icahn School of Medicine at Mount Sinai, New York, NY 10029-6574, USA; 16 Research Institute, Fundación Universitaria de Ciencias de la Salud-FUCS, Bogotá 11011, Colombia; 17 Servicios y Asesorías en Infectología-SAI, Bogotá 11010, Colombia; 18 Gilbert and Rose-Marie Chagoury School of Medicine, Lebanese American University, Beirut P.O. Box 36, Lebanon

**Keywords:** *Anaplasma phagocytophilum*, *Anaplasma platys*, *Ehrlichia canis*, *Ehrlichia ewingii*, *Dirofilaria immitis*, complete blood counts, tick-borne diseases, hemothropic pathogens, canine, zoonotic, Latin America

## Abstract

**Simple Summary:**

Canine ehrlichiosis, anaplasmosis, and other vector-borne diseases are relevant for public health in the tropics. Despite this, studies characterizing certain clinical aspects, such as haematological alterations, are lacking, especially in Colombia and Latin America. In the present study, in an area where previously no studies on such diseases have been published, we identified and characterized, by serological and molecular testing, dogs with such infections to describe and compare their haematological alterations according to the infection status. That led to the finding that thrombocytopenia, neutropenia, and pancytopenia, are significant features of infection, even with a high positive predictive value. That highlights the importance of haematology in the suspicion of ehrlichiosis, anaplasmosis and other vector-borne diseases, especially in endemic areas.

**Abstract:**

Infections due to *Ehrlichia*, *Anaplasma*, *Dirofilaria*, *Mycoplasma*, *Babesia* and *Hepatozoon* continue to be highly prevalent in dogs, especially in tropical and subtropical areas, where vectors of many of them are present. However, many clinical aspects of dogs have not been characterized in detail, including assessing the haematological alterations associated with them, particularly in Colombia and Latin America. A group of 100 dogs with *Ehrlichia*, *Anaplasma*, *Dirofilaria*, *Mycoplasma*, *Babesia* and *Hepatozoon* infections/exposure were assessed by blood smear serology (SNAP4DX) and PCR in Pereira, Colombia. We performed blood counts to evaluate anaemia, leukopenia/leukocytosis, neutropenia, neutrophilia, lymphopenia/lymphocytosis, monocytosis, eosinophilia, and thrombocytopenia, among other alterations. Bivariate analyses were performed on Stata^®^14, with significant *p* < 0.05. From the total, 85% presented ≥1 infection (past or present), 66% with coinfections (≥2 pathogens) (*Ehrlichia* 75%), and 89% presented clinical alterations. A total of 100% showed anaemia, 70% thrombocytopenia, 61% monocytosis, and 47% neutropenia, among other alterations. Additionally, 11% presented pancytopenia and 59% bicytopenia. The median platelet count was lower in infected dogs (126,000 cells/μL) versus non-infected (221,000 cells/μL) (*p* = 0.003). Thrombocytopenia was higher among infected dogs (75%) versus non-infected (40%) (*p* = 0.006), with a 91% positive predictive value for infection. Median neutrophil count was lower in infected dogs (6591 cells/μL) versus non-infected (8804 cells/μL) (*p* = 0.013). Lymphocytosis occurred only among those infected (27%) (*p* = 0.022). Leukopenia was only observed among infected dogs (13%). Pancytopenia was only observed among infected dogs. Ehrlichiosis and other hematic infections have led to a significant burden of haematological alterations on infected dogs, including pancytopenia in a tenth of them, most with thrombocytopenia and all anemic.

## 1. Introduction

Tick-borne diseases are a significant problem for the health of canines, and many of them are also zoonotic and risky for humans. Diseases such as anaplasmosis, ehrlichiosis, and babesiosis, among others, are still of limited knowledge globally, as well as in those highly endemic areas, such as Latin America, including countries such as Colombia, both in humans and in canines.

For example, few investigations on babesiosis have been carried out in Colombia, except in the Colombian Caribbean region and Urabá, Antioquia [1,2,3]. Babesiosis may cause febrile syndrome in humans and infection in dogs, leading to considerable morbidity and the possibility of fatal outcomes in both, especially in those with comorbidities and risk factors [4]. Such hemoparasitoses, not only babesiosis, are caused by organisms from various etiological agents, mainly protozoa, bacteria of the order Rickettsiales, and spirochetes. Consequently, they can live inside red blood cells or other blood cells and cause anaemia. In the case of *Anaplasma* and *Ehrlichia* infections, there are few studies in humans and limited studies in dogs [5,6,7,8,9,10,11,12,13]. Furthermore, in the case of Colombia and Latin America in general, but even globally, as mentioned, few studies have characterized, in detail, the profile of haematological alterations in canines with hemoparasitic infections, such as those mentioned, which would be helpful for veterinarians, especially in endemic areas.

When reviewing the international literature, it appears that all studies aimed at providing information related to the hematological profile associated with these pathogens’ infection may still be of significant importance [1,2,3].

Despite the epidemiological importance that the different infections caused by hemoparasites in canines have had during the last few decades in the world, to date, there are relatively few studies in the world literature, even more so in Colombia, that have characterized the profile of the haematological alterations observed in canines with said infections [5,6,7,8,9,10,11,12,13]. Anaemia, thrombocytopenia, and variable cellular responses from leukopenia to lymphocytosis and monocytosis have been described in dogs with ehrlichiosis. In some canines, the microorganism can be eliminated, although in the majority it persists, establishing the chronic phase, especially when not treated appropriately [14]. This infection manifests as a mild disease with haematological alterations and weight loss but also deterioration of marrow production, which later causes even pancytopenia. Such immune system compromise is ineffective for controlling the microorganisms, leading to fatal outcomes in the animal [15]. In the case of babesiosis, less virulent *Babesia* species and strains typically cause hemolytic anaemia and pyrexia; intraerythrocytic parasitaemia causes both intravascular and extravascular hemolysis, resulting in anaemia, hemoglobinuria, bilirubinuria, and hemoglobinemia [15,16].

Consequently, anaemia is produced by the lysis of erythrocytes, which are destroyed by the exit of the parasites but also favour their development by increasing erythrocyte fragility and erythrophagocytosis [17]. In anaplasmosis, the microorganism, once located within the bloodstream, seeks out white blood cells and enters via endocytosis [18,19], forming vacuoles, where it multiplies by binary fission to form up to eight organisms; these leave the erythrocyte by exocytosis and infect the surrounding erythrocytes [20], which implies that there is no destruction of the infected red blood cells and therefore there is no severe anaemia as in other hemoparasitoses. However, despite the above, not many studies indicate the frequency of those alterations, such as thrombocytopenia, anaemia, leukocytosis, and leukopenia.

Multiple studies on vector-borne (VB) infections have been carried out in humans and dogs globally. However, there are not enough data about the haematological alterations associated with infections due to *Ehrlichia*, *Anaplasma*, *Babesia*, *Dirofilaria*, *Mycoplasma*, *Hepatozoon* and other VB infections in dogs.

Herein, we present a prospective study to assess such haematological alterations in dogs with confirmed infection/exposure due to *Ehrlichia*, *Anaplasma*, *Babesia*, *Dirofilaria*, *Mycoplasma*, *Hepatozoon* and other hematic organisms amongst dogs inhabiting the municipality of Pereira, Risaralda department, Colombia, in 2020.

## 2. Materials and Methods

### 2.1. Study Area

**Figure 1:**
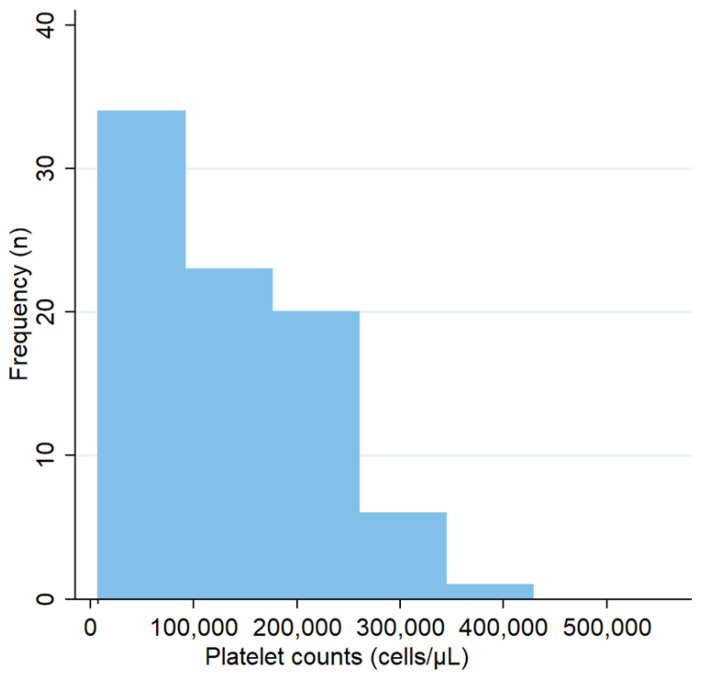
Frequency distribution of the platelet counts among infected dogs.

Pereira is the principal city of the Coffee Triangle region, which includes three departments (first administrative territory level) and 53 municipalities (second administrative territory level). Pereira is the capital of Risaralda (967,780 habitants in 2020), a department surrounded by six other western departments (Antioquia, Caldas, Tolima, Quindio, Valle del Cauca and Chocó) [21]. Pereira’s landscape embraces both urban and rural areas. The first consists of 20 communities (the city) and the second of 12 corregiments (sub-municipalities) (both tertiary administrative territory level) (Figure 1). According to the National Statistics Department (DANE, www.dane.gov.co, accessed on 1 January 2021), the total population for 2020 reached 477,027 inhabitants. The metropolitan area includes Dosquebradas and La Virginia municipalities, with 709,338 inhabitants in 2020. Rio Otún, Centro, San Joaquin, Del Café, Boston, El Oso, Consotá and Cuba are the most populated areas of the Pereira municipality, making up 51% of its population. The city of Pereira extends over an area of 702 Km^2^ (4°48′51″ N 75°41′40″ W). The climate is tropical, with an annual median temperature of 18.8 °C (median minimum of 15.8 °C, median maximum of 26.3 °C).

### 2.2. Animals

Based on the municipality dog census, we calculated a minimum sample of 98 dogs to be assessed. Finally, 100 with suspected infection were distributed in four urban communes (out of 20) and four rural corregiments (out of 12) of the municipality Pereira and its neighbouring municipality of Dosquebradas. Only one dog was included from those owners who voluntarily agreed to participate with their dogs, regardless of where co-living was in the same house. Dogs presenting fever, swollen lymph nodes, respiratory distress, weight loss, bleeding disorders (spontaneous haemorrhage or bleeding), neurological disturbances, anaemia, and the presence of ticks, among others, were considered to have suspicion of infection and were included.

### 2.3. Collection of Blood Samples

Blood samples from each dog were individually collected from the radial vein into a sterile vacuum tube (Vacutainer, Becton, Dickinson and Company Franklin Lakes, Franklin Lakes, NJ, USA).

### 2.4. Samples Analysis

To determine selected vector-borne pathogens exposure, a rapid enzyme-linked immunosorbent assay (ELISA) kit (SNAP^®^ 4Dx^®^ Plus Test Kit, IDEXX Laboratories, Inc, Westbrook, ME, USA) was used, following the manufacturer’s instructions [16]. This qualitative test allowed us to simultaneously detect the presence of circulating antibodies (IgG and IgM) against immunodominant proteins of *E. canis*/*ewingii* (p30 and p30-1, sensitivity of 96.2%), *A. phagocytophilum*/*platys* (p44/MSP2, sensitivity of 99.1%), *B. burgdorferi* s.l. (C6, the sensitivity of 98.8%), and *D. immitis* antigens (principally produced by adult females) based on specific antibodies (sensitivity of 99.2%). The SNAP^®^ 4Dx^®^ Plus Test Kit showed a specificity of ~100% for the microorganisms mentioned above [22,23,24]. 4Dx^®^ Plus Test Kit has been validated in dogs [22,23,24]. As part of this project, seroprevalence in the area has been presented elsewhere [25].

Blood samples (1 mL w/EDTA) were tested using a standardized qPCR for the detection of DNA of *Anaplasma* spp. (Ricketsiales 16S rRNA, 345 kbp), *Ehrlichia* spp. (Ricketsiales 16S rRNA, 345 kbp), *Hepatozoon* spp. (18S rRNA, 227 kbp), *Dirofilaria* spp. (12S rRNA, 90 kbp), *Babesia* spp. (18S rRNA, 207 kbp), and *Mycoplasma* spp. (16S rRNA, 193 kbp).

Finally, we performed complete blood counts to assess anaemia, leukopenia/leukocytosis, neutropenia, neutrophilia, lymphopenia/lymphocytosis, monocytosis, eosinophilia, and thrombocytopenia, among other alterations. From blood samples (3 mL) of evaluated canines, peripheral blood smears were made, which were prepared with a variant of the Romanowsky stain (Diff-QuikTM) at the laboratory for observation under a light microscope (100×) (Scientific^®^), with a sensitivity of 92% for specific organisms, depending on their bacterial/parasitic load in blood [26]. With that, possible infection in blood cells, such as erythrocytes, leukocytes and platelets, was evidenced at different stages of infectious agents [27]. Of the peripheral blood samples, 13 µL were used to perform a complete blood count or haematology analysis on a Mindray BC-2800 Vet^®^ equipment (Guangdong Sheng, China), which uses electrical impedance for erythrocyte, leukocyte, and platelet counting and the SFT method for haemoglobin. It is an automatic 3-part haematology analyzer with 13 parameters for blood cell count tests and microsampling technology, with predefined and standardized settings for canine populations. The equipment allows analysis of the following haematological variables: total count of white cells or leukocytes (WBC), number of lymphocytes (Lymph#), number of granulocytes (Gran#), percentage of lymphocytes (Lymph %), mean cell percentage (Mid%), granulocyte percentage (Gran %), red blood cell count (RBC), haemoglobin (HGB), hematocrit (HCT), mean corpuscular volume (MCV), mean corpuscular haemoglobin (MCH), mean corpuscular haemoglobin concentration (MCHC), and platelet count (PLT). When analyzing, the equipment’s recommended reference values and those recommended internationally were considered [28]. The measurement precision of the equipment is for leukocytes, 3 (coefficient of variation, CV%, 4.0–15.0); erythrocytes, 2 (3.00–6.50); haemoglobin, 2 (100–180); corpuscular volume medium, 1 (70.0–110.0); and platelets, 5 (200–500). These haematological variables were analyzed in their natural state and categorized.

### 2.5. Statistical Analysis

Statistical analysis was performed using Stata 14^®^IC (Stata Corp., College Station, Texas, USA). Chi-square tests were used to compare the proportions of positivity related to categorical dependent variables and establish statistical significance. Median values and interquartile ranges were calculated for the haematological variables. A Wilcoxon signed-rank test was used to compare quantitative haematological variables. We estimated the sensitivity, specificity, and positive and negative predictive values (with their respective 95% confidence intervals) for thrombocytopenia for infection. A *p* < 0.05 was considered statistically significant.

## 3. Results

The mean age of the canine population was 4.2 years (± 2.89 years, range 0.21–12.56), 53% were female, and 47% were males. The average age did not differ significantly by sex (males 4.34 ± 3.27 years; females 4.10 ± 2.59 years) (*p* = 0.6345). Other dog variables were previously presented [19]. 

From the total, 85% presented ≥1 infection (past or present, by PCR or SNAP^®^ 4Dx^®^, 53% were positive at both), 66% with coinfections (≥ 2 pathogens), corresponding to *Ehrlichia* spp. in 75%, *Mycoplasma* spp. 33%, *Dirofilaria* 28%, *Hepatozoon* spp. 28%, *Anaplasma* spp. 16%, *Babesia* spp. 15%, and 0% *Borrelia* spp.; 89% presented clinical alterations. Table 1 shows the median of the haematological values and their corresponding interquartile ranges.

The most frequent alteration in all dogs was anaemia (100%) (Table 1), followed by thrombocytopenia (70%) (13% had severe thrombocytopenia, <35,000 platelets/μL), monocytosis (61%), neutropenia (47%), leukocytosis (23%), lymphocytosis (23%), lymphopenia (16%), neutrophilia (14%), leukopenia (11%), and eosinophilia (10%). Additionally, 11% presented pancytopenia, and 59% was bicytopenia (Table 1).

**Figure 2:**
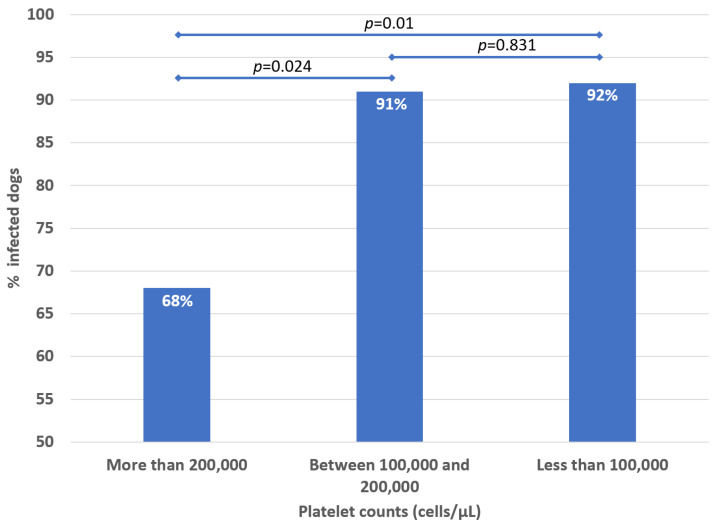
Proportion of infected dogs according to the categories of platelet counts (*p*-values comparing the categories were included, *p* < 0.05, considered significant differences in the proportion of infected dogs by categories of platelet counts).

The median platelet in the whole group of dogs was 143,000 cells/μL (IQR 62,750–214,250), being significantly lower in the infected dogs (126,000 cells/μL, IQR 55,000–191,000) (Figure 1) versus non-infected (221,000 cells/μL, IQR 180,000–303,500) (*p* = 0.003) (Table 1). Thrombocytopenia was higher among infected dogs (75%) versus non-infected (40%) (*p* = 0.006) (14% of the infected dogs had severe thrombocytopenia, while 7% of the not-infected dogs). At the same time, the proportion of infected dogs was significantly higher among those with lower levels of platelets (*p* < 0.05), especially below 200,000 cells/μL (Figure 2).

Median neutrophil count was lower in infected dogs (6591 cells/μL, IQR 4806–9112) versus non-infected (8804 cells/μL, IQR 6677–13,555) (*p* = 0.013). Neutropenia was higher among infected dogs (51%) versus non-infected (27%) (*p* = 0.087). Lymphocytosis occurred only among those infected (27%) (*p* = 0.022). Leukopenia was only observed among infected dogs (13%) (Table 1). Bicytopenia was higher among infected dogs (62%) than non-infected (40%) (*p* = 0.105). Pancytopenia was only observed among infected dogs (Table 1). Other comparisons were not significant (*p* ≥ 0.05), although, in many of the variables, alterations were higher among those infected (Table 1). Sex and age did not significantly impact the results.

Regarding the platelet counts, it was found that comparing by sex (Table 2), the levels were significantly lower among infected male dogs (median 136,500 cells/μL) than in non-infected male dogs (median 281,000 cells/μL) (*p* = 0.003). Consequently, thrombocytopenia was also significantly higher in those infected male dogs (74%) than in those non-infected male dogs (20%) (*p* = 0.015) (Table 2). On the other hand, no significant differences were found for females (*p* ≥ 0.05) (Table 2).

The sensibility of thrombocytopenia for infection was 75.29% (95% Cl 65.54–85.05), with a specificity of 60.0% (95% Cl 31.87–88.13). Its positive predictive value was 91.43% (95% Cl 84.16–98.70), and its negative predictive value of 30% (95% Cl 11.94–48.06).

**Figure 3:**
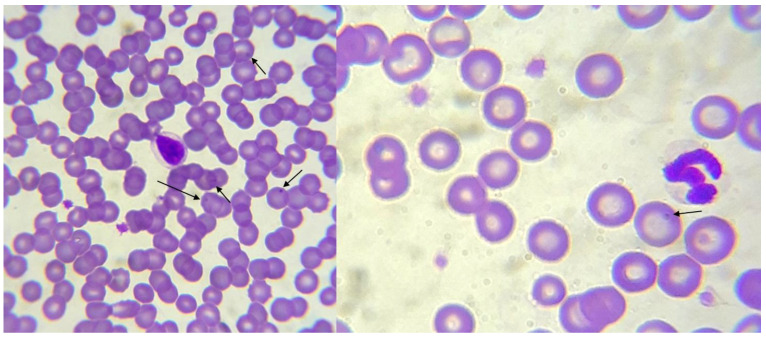
Blood smear from a dog with suspicion of *Anaplasma* that appears as a small and roughly spherical intraerythrocytic parasite (arrows), Wright–Giemsa staining (1000×). Arrows indicate the suggestive forms.

**Figure 4:**
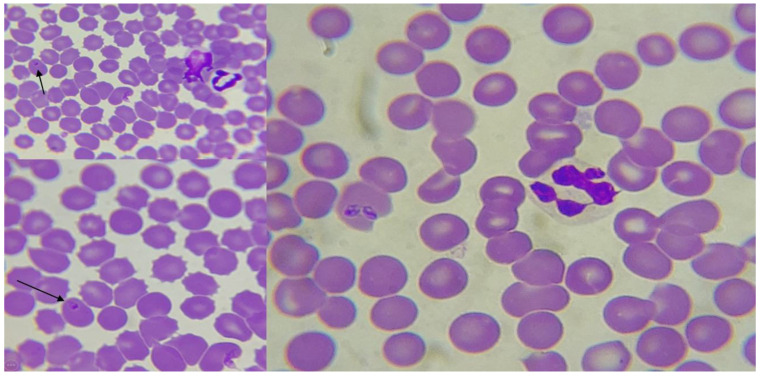
Blood smear from a dog suspected *Babesia* infection, average size red blood cells with intraerythrocytic ring forms, Wright–Giemsa staining (1000×). Arrows indicate the suggestive forms.

At blood smears in five cases, images were suggestive of *Anaplasma* (three cases) (Figure 3) and *Babesia* (two cases) (Figure 4), and *Theileria* among other organisms (Table 1).

## 4. Discussion

Canine vector-borne diseases continue to be a significant public health threat, as many of them are the same zoonotic. However, from the clinical perspective of such infections, there is a lack of studies developing proper characterizations of the haematological alterations among dogs with infections due to pathogens such as *Ehrlichia, Anaplasma, and Babesia*, among others. These hemotropic organisms can significantly impact different blood cells, with their consequent clinical implications. Therefore, suspicion of such infections, based on haematological alterations, is vital, especially in tropical, subtropical and endemic areas, as is the case of Colombia and other countries of Latin America. In this study, thrombocytopenia, low levels of neutrophils, lymphocytosis, and pancytopenia were found markedly among those infected. Additionally, all the infected and non-infected dogs had anaemia.

In a recent study from Portugal, Spain, and Italy, retrospectively reviewing 21 records of dogs with PCR-confirmed *Anaplasma platys* infection, complete blood counts were available, found anaemia and thrombocytopenia in 81%, leukocytosis 33.3%, and leukopenia 23.8% [29]. In that study, 33.3% of the dogs had severe thrombocytopenia, while in ours, this was 13% (14% among those infected). In that assessment in the Mediterranean basin, leukocytosis was due to neutrophilia in all seven cases, whereas leucopenia was due to neutropenia in all five cases. Similarly, low neutrophil counts were significantly higher in our study among infected dogs. In that evaluation, authors found five dogs with pancytopenia (29.4%), while in our study, this was in 13% of the infected dogs.

In another study from Germany, which included 18 dogs with anaplasmosis, 16 dogs (89%) were found to have thrombocytopenia, 10 (56%) anaemia, 5 (28%) leukopenia, 9 (50%) lymphopenia, 4 (22%) leukopenia, 1 (6%) eosinophilia, 2 (11%) lymphocytosis, among other alterations [30]. However, in that study, pancytopenia was not reported. Furthermore, in that study, neutropenia was reported in 13%, while in our study, among 51% of the infected dogs.

A study in Greece, among 19 dogs infected with *Ehrlichia canis*, found that all of them presented anaemia and thrombocytopenia (100%), and in 13 dogs (68%), severe thrombocytopenia was observed [31]. In addition, the authors also found lymphopenia (95%), neutropenia (90%), and pancytopenia (90%).

Another study in Greece, retrospective, assessing medical records of 850 client-owned dogs, found that thrombocytopenia was significantly higher (66%) among those with positive anti-*E. canis* IgG antibodies, compared to those negative (38%) (*p* < 0.0001) [32]. In addition, anaemia and pancytopenia (57% and 8%) were significantly higher among those infected than in those free of the pathogen (36% and 3%, respectively) (*p* < 0.01).

A study among 73 seropositive dogs (for *E. canis*) in India found that 100% presented thrombocytopenia [33]. Another study in the same country, with 46 dogs with ehrlichiosis, reported significantly lower levels of platelets than their controls (*p* < 0.001) and also of haemoglobin and erythrocytes (*p* < 0.05) [34].

Haematological abnormalities have been reported in isolated cases of anaplasmosis and ehrlichiosis [35,36], but there is a lack of studies in this regard, especially in Latin America. Moreover, for ehrlichiosis, babesiosis and other hemoparasitic infections, there are few or no studies in the international literature on this matter.

In a study in Brazil, in 217 dogs, it was found that in those with thrombocytopenia of fewer than 100,000 platelets/mL, 63.1% were infected with *Ehrlichia canis*; in those with 100,000–200,000 platelets/mL, 21% were infected and in those without thrombocytopenia, only 1.4% were infected with *E. canis* [37]. As observed in that study in Brazil, there is an inverse relationship between the frequency of infection according to the platelet count, which was also observed in our study (Figure 2). The different phases of disease would also explain this intensity. Although our study is cross-sectional, cohort studies are necessary to characterize the evolution of thrombocytopenia among infected dogs.

A study with 73 dogs in Thailand found that the frequency of hematocrit values <15% was significantly higher in those infected with *E. canis* (31%) compared to those without infection (5.9%) (*p* < 0.05) [38]. However, none of these studies detailed the differences in thrombocytopenia or other parameters by sex of the dogs [32,37], and we found in the present study that thrombocytopenia was significantly higher among infected male dogs (75%) than in non-infected males (20%).

Regarding the usefulness of thrombocytopenia, there is a lack of studies on the sensibility, specificity, and positive and negative predictive values for infection [39,40,41]. In this study, we found that thrombocytopenia has a good sensibility for the infection (75%) but a high positive predictive value (91%), which is very useful in endemic areas where the prevalence of ehrlichiosis, anaplasmosis would be high, as previously reported in Pereira, Colombia (74%) [25].

Platelet consumption increased splenic sequestration, and decreased platelet lifespan are the possible attributes of thrombocytopenia [34,42]. Depleted synthesis of coagulation proteins in the liver due to necrosis may also cause increased platelet aggregation, further aggravating thrombocytopenia [34,43]. Thrombocytopenia, mainly due to large-scale destruction of the cells in the spleen, that begins a few days after the infection [34,44] alongside bone marrow hypoplasia, is also amongst the primary causes of pancytopenia, including thrombocytopenia [34].

As can be seen, there is a need to characterize better the profile of haematological alterations that occur in dogs with hemotropic infections. That can be useful in specifically suspecting hematic infections and helping their initial differentiation. In the descriptions of the infections by these organisms, they are not necessarily sufficiently well-characterized or described by which the present work presents to characterize them, given the lack of existing information in this regard. Laboratory findings such as anaemia, leukopenia and thrombocytopaenia must be included in the differential diagnosis when observed during routine laboratory evaluations for diagnosis of canine ehrlichiosis and anaplasmosis [34]. In addition, in future studies with larger samples would be interesting to assess if differences in the haematological alterations may be influenced by the age and sex of the dogs; however, in this study, we did not find significant differences, although infected female dogs presented the lower levels of platelets count, and the highest relative frequency of thrombocytopenia (77%).

Although our study has more samples than others previously reported, we should acknowledge as a limitation that this study only assessed 100 dogs, preventing the possibility of running multivariate and other analyses, as well as to compare by each specific etiological agent.

## 5. Conclusions

Canine vector-borne diseases, including ehrlichiosis and other haematic infections, have led to a significant burden of haematological alterations on infected dogs, including pancytopenia in a tenth of them, most with thrombocytopenia and all anaemic, in addition to other significant alterations. Given these findings, especially relevant for places with a lack of laboratory tests for infection, even in areas with a lack of PCR or serological tests, the haematological assessment is critical in the suspicion of disease, especially considering its high positive predictive value, especially in endemic areas such as Pereira and other zones of Colombia and Latin America.

## Figures and Tables

**Table 1:** Summary of the haematological variables among dogs, infected and non-infected.

	Total (n = 100)			Infected Dogs (n = 85)			Non-Infected Dogs (n = 15)			
Haematological Variables	Median Values	IQR		Median Values	IQR		Median Values	IQR		*p*-Value *
Hematocrit (%) (RV ≥ 38)	39.65	34.70	45.35	39.5	34.3	45.9	40.70	38.70	42.75	0.602
Hemoglobin (g/dL) (RV ≥ 14)	29.60	29.20	30.33	29.6	29.2	30.2	30.40	29.40	30.75	0.074
Red blood cells (×10^6^/μL) (RV ≥ 5.5)	7.18	6.33	8.09	7.11	6	8	7.42	6.83	7.58	0.938
Mean corpuscular volume (MCV) (RV ≥ 62)	55.60	53.60	58.33	56	53	59	56.40	54.90	57.65	0.409
% with low MCV	96			96			93			0.568
Mean corpuscular haemoglobin (MCH) (RV ≥ 22)	16.65	15.80	17.40	17	16	17	16.90	16.60	17.65	0.309
% with low MCH	98			98			100			0.548
Mean corpuscular haemoglobin concentration (MCHC) (RV ≥ 32)	29.60	29.20	30.33	30	29	30	30.40	29.40	30.75	0.075
% with low MCHC	97			96			100			0.460
% with anaemia	100			100			100			N/A
White blood cells (×10^9^/L) (RV 6000–17,000)	12,550	8900	16,250	12,000	8200	16,000	13,500	11,850	19,050	0.087
% with leukopenia	11			**13**			0			0.140
% with leukocytosis	23			20			40			0.090
Neutrophil count (×10^9^/L)	6903	4986	9517	6591	4806	9112	8804	6677	13,555	**0.013**
% of neutrophils (RV 60–70)	61	50	68	59	49	68	66	58.5	70.5	**0.044**
% with neutropenia	47			51			27			0.087
% with neutrophilia	14			12			27			0.125
% of lymphocytes (RV 12–30)	20	14	30	20	14	32	18	12	22.5	0.161
% with lymphopenia	16			14			27			0.222
% with lymphocytosis	23			**27**			**0**			**0.022**
% of monocytes (RV 3–10)	15	9	19.25	15	9	20	11	9.5	18.5	0.667
% with monocytopenia	2			1			7			0.161
% with monocytosis	61			62			53			0.509
% of eosinophils (RV ≤ 10)	3	1	7	3	1	7	3	1	7	0.911
% with eosinophilia	10			8			20			0.161
% of basophils	0	0	0	0	0	0	0	0	0	N/A
% of reticulocytes	0	0	0	0	0	0	0	0	0	N/A
Ortochromatic normocites	2	1	2	3	1	2	0	0	0	N/A
Platelet counts (cells/μL) (RV ≥ 200,000) **	143,000	62,750	214,250	**126,000**	55,000	191,000	**221,000**	180,000	303,500	**0.003**
% with thrombocytopenia	70			**75**			**40**			**0.006**
% with severe thrombocytopenia (<35,000 platelets/μL)	13			14			7			0.429
% with macroplatelets	13			19			13			0.470
% with anisocytosis	11			16			0			0.336
% with hypochromia	10			18			7			0.758
% with polychromacy	8			9			7			0.913
% with poikilocytosis	4			4			7			0.568
% with toxic neutrophils	4			7			0			0.692
% with reactive lymphocytes	2			1			7			0.161
% with agglutination	1			2			0			0.673
% with spherocytes	0			0			0			N/A
% with bicytopenia	59			62			40			0.105
% with pancytopenia	11			13			0			0.140
Total proteins (g/dL) (RV ≥ 5.5)	10.50	9.88	11.28	10.5	10	12	9.50	8.25	10.25	**0.003**
% with hypoproteinemia	1			1			0			0.673
% with organisms in the blood smear	5			6			0			N/A
Suggestive of *Anaplasma marginale*, marginal at erythrocytes	2			2			0			N/A
Suggestive of *Babesia*	2			2			0			N/A
Suggestive of *Anaplasma*, pyriform inclusions, Howell–Jolly bodies	1			1			0			N/A
Suggestive of *Theileria*	1			1			0			N/A

IQR, interquartile range. * Comparing infected versus not infected. RV, reference value. ** Severe thrombocytopenia was considered with <35,000. Bold, statistically significant (*p* < 0.05).

**Table 2:** Sex comparisons for platelet counts and thrombocytopenia according to infection status.

		Infected Dogs			*p* (Male vs. Female)	Non-Infected Dogs			*p* (Male vs. Female)	*p* (Infected vs. Non-Infected by Sex)
	Sex	Median Value	IQR			**Median Values**	**IQR**			
Platelet counts (cells/μL)	Male	**136,500**	48,250	197,750	0.705	**281,000**	236,000	320,000	0.270	**0.003**
	Female	105,000	64,500	178,000		206,000	106,750	270,500		0.104
			**%**		***p*** **(Male vs. Female)**		**%**		***p*** **(Male vs. Female)**	***p*** **(Infected vs. Non-Infected by Sex)**
		**n**	**Yes**	**No**		**n**	**Yes**	**No**		
Thrombocytopenia	Male	42	**74**	26	0.754	5	**20**	80	0.264	**0.015**
	Female	43	77	23		10	50	50		0.091

IQR, interquartile range. Bold, statistically significant (*p* < 0.05).

## Data Availability

Available upon reasonable request.
